# Supplementary figures and images for: Muscle-resident mesenchymal progenitors sense and repair peripheral nerve injury via the GDNF-BDNF axis
Source: eLife. 2024 Sep 26;13:RP97662. doi: 10.7554/eLife.97662 (PMC11426970; doi:10.7554/eLife.97662)

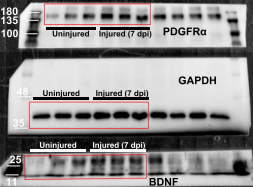

Supplement: Figure 5—source data 1. [file elife-97662-fig5-data1.zip › merged_with_marker.pdf]

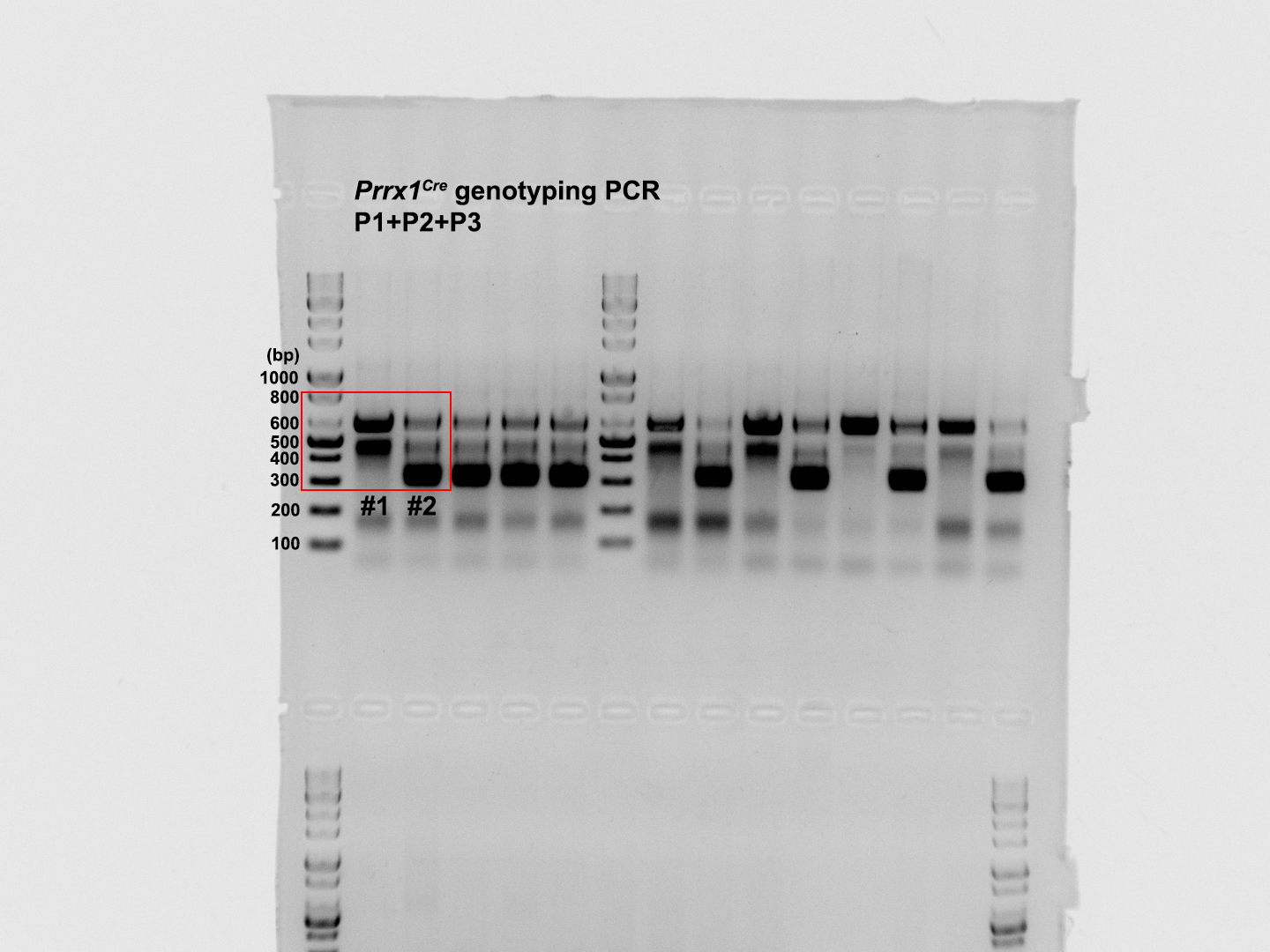

Supplement: Figure 6—figure supplement 1—source data 1. [file elife-97662-fig6-figsupp1-data1.zip › Figure 5-figure supplement 1B_left panel_labeled.tif]

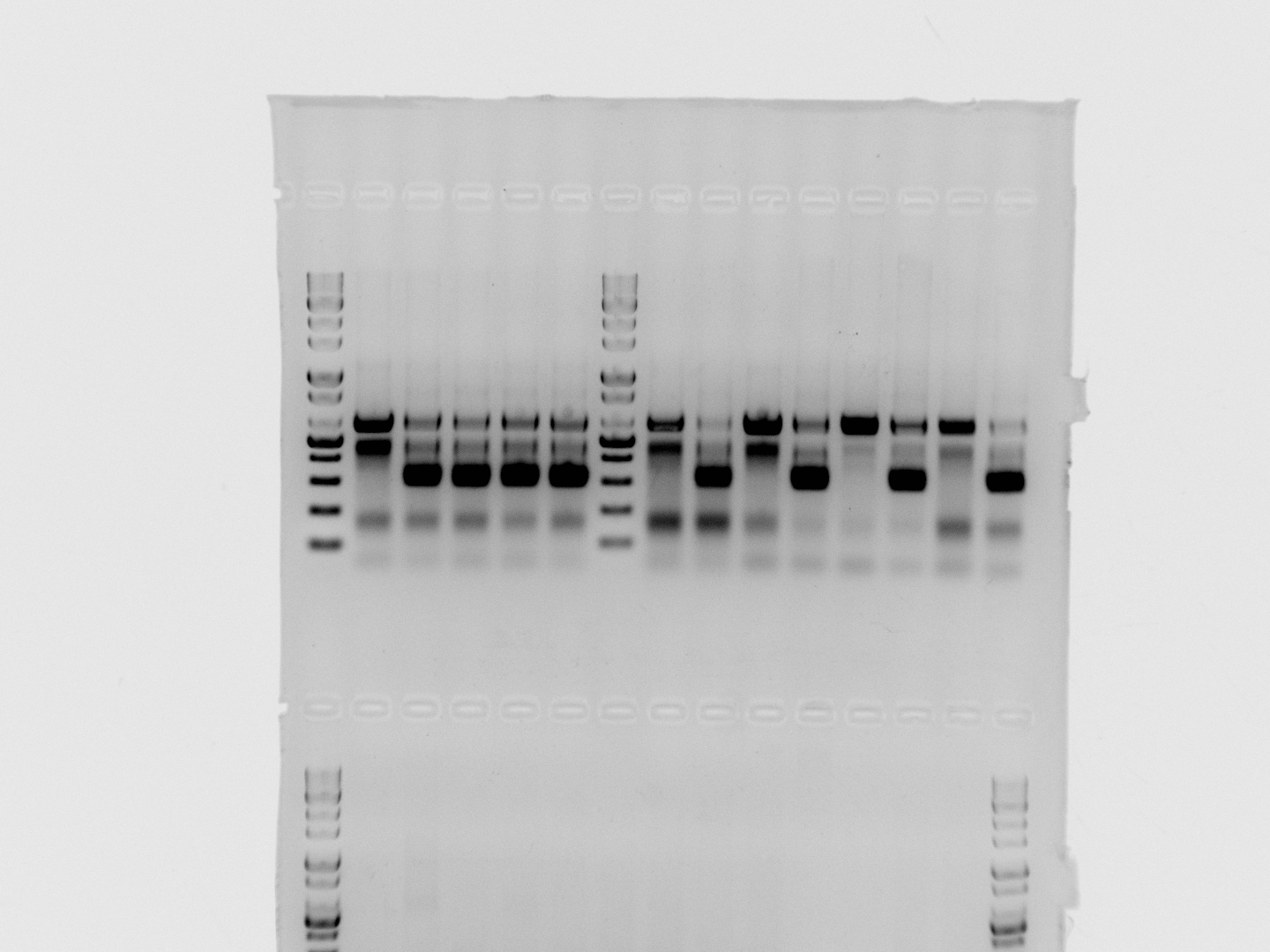

Supplement: Figure 6—figure supplement 1—source data 1. [file elife-97662-fig6-figsupp1-data1.zip › Figure 5-figure supplement 1B_left panel_raw.tif]

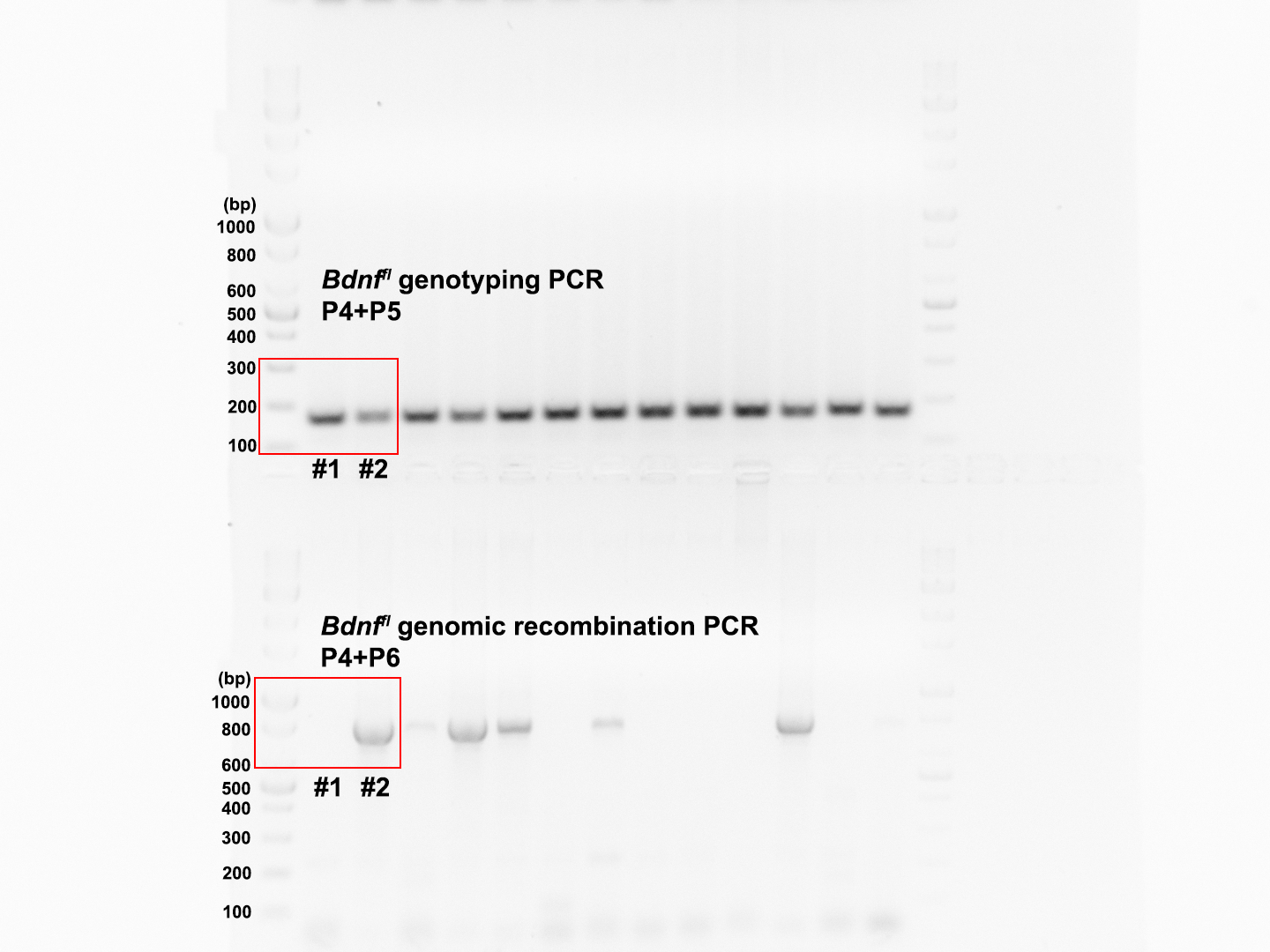

Supplement: Figure 6—figure supplement 1—source data 1. [file elife-97662-fig6-figsupp1-data1.zip › Figure 5-figure supplement 1B_middle and right panel_labeled.tif]

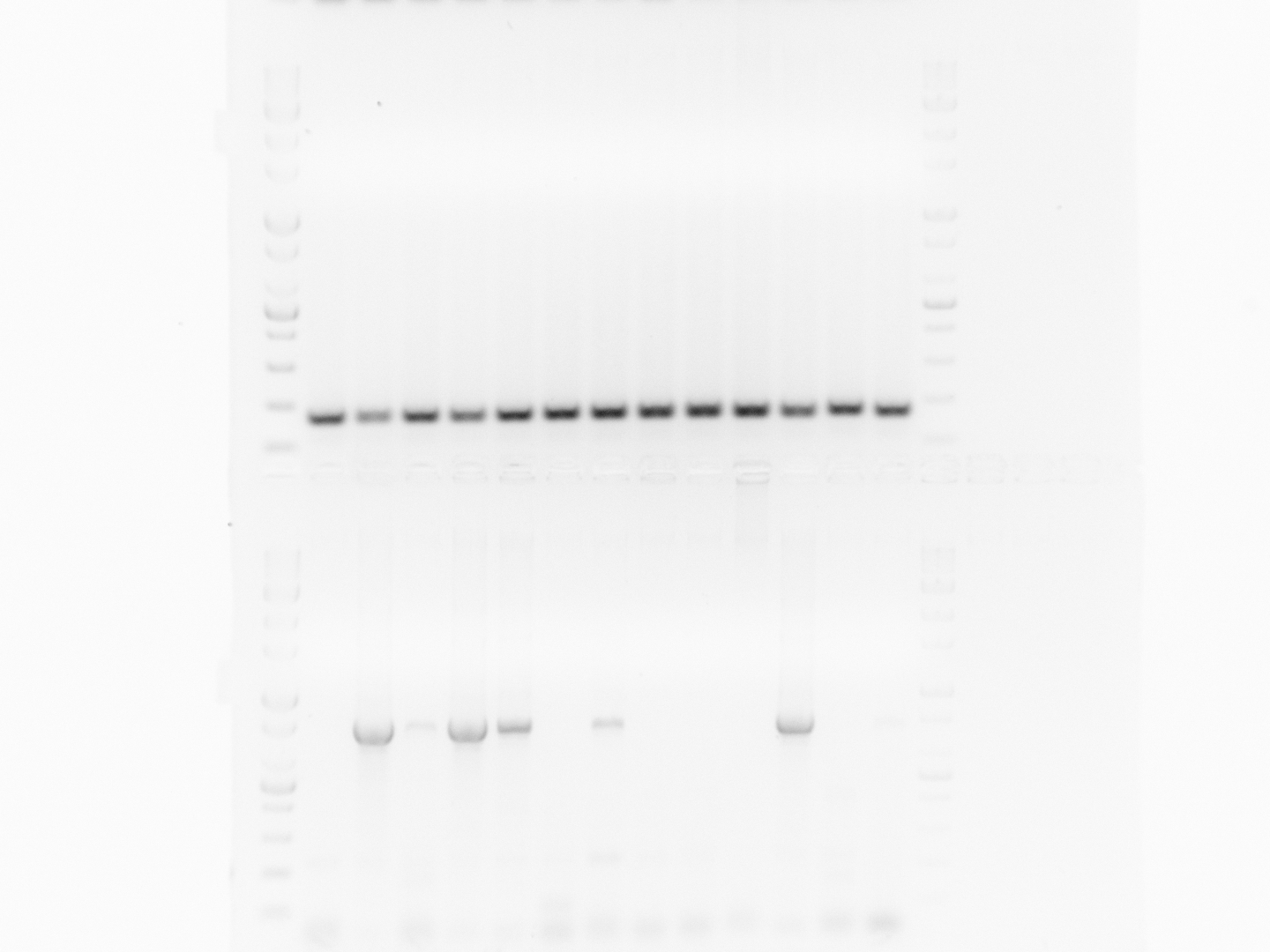

Supplement: Figure 6—figure supplement 1—source data 1. [file elife-97662-fig6-figsupp1-data1.zip › Figure 5-figure supplement 1B_middle and right panel_raw.tif]
