# Supplementary material for: Muscle-resident mesenchymal progenitors sense and repair peripheral nerve injury via the GDNF-BDNF axis
Source: eLife. 2024 Sep 26;13:RP97662. doi: 10.7554/eLife.97662 (PMC11426970; doi:10.7554/eLife.97662)
Supplement: Supplementary file 1. — (a) Results from TRRUST show transcription factors predicted to regulate genes specifically enriched in cluster 2. Genes known to be regulated by each transcription factor is listed. (b) Results from TRRUST showing transcription factors predicted to regulate genes specifically enriched in cluster 3. Genes known to be regulated by each transcription factor is listed. (c) Categorization of genes predicted to be regulated by transcription factors that act downstream of the glial cell line-derived neurotrophic factor (GDNF) signaling pathway. Note that only Bdnf fits into all three criteria. (d) Primers used in this study for genotyping PCR or RT-qPCR. [file elife-97662-supp1.docx]

**Supplementary file 1a.**

| **Key TF** | **Adjusted *P* value** | **List of overlapped genes** |
| --- | --- | --- |
| Jun | 0.000114 | Timp1, C1qtnf3, Plaur, Slc39a14, Mmp3, Itga5, Serpine1, Tgfb1, Mmp19, Ccl2, Slc8a1, Plau |
| Smad3 | 0.000399 | Serpine1, Plau, Nedd9, Acta2, Ccl2, Tagln, Enpp1 |
| Msx2 | 0.00464 | Lss, Enpp1, Tagln, Alpl |
| Smad1 | 0.00579 | Runx1, Col4a1, Col4a2, Tnfrsf11b |
| Ppard | 0.00579 | Angptl4, Tgfb1, Insig1 |
| Sp1 | 0.00579 | Acta2, Trf, Serpine1, Plaur, Lgals1, Mgp, Ccl2, Nes, Adcyap1r1, Itga5, Bmp4, Notch1, Skil |
| Fos | 0.0149 | Tgfb1, Bdnf, Tslp, Plau, Pdpn |
| Tgfb1i1 | 0.0149 | Ccl2, Tgfb1 |
| Srf | 0.0185 | Tagln, Barx2, Acta2 |
| Id3 | 0.0298 | Notch1, Alpl |
| Nfkb1 | 0.0298 | Mmp3, Tgfb1, Dmp1, Pla2g4a, Il15, Egr2, Plaur, Ppp1r13l, Ccl2 |
| Junb | 0.034 | Plau, Serpine1 |
| Pparg | 0.034 | Serpine1, Muc1, Angptl4, C1qtnf3 |
| Nfkb2 | 0.0362 | Hif1a, Dmp1 |
| Id1 | 0.0382 | Thbs1, Alpl |
| Runx2 | 0.0382 | Alpl, Mgp, Thbs1, Enpp1 |
| Arntl | 0.0382 | Cxcl5, Bhlhe41 |
| Msx1 | 0.0382 | Tagln, Bmp4 |
| Nr4a2 | 0.0382 | Tnc, Gch1 |
| Ets1 | 0.0388 | Timp1, Mmp3, Ccl2, Bmp4 |
| Prox1 | 0.0465 | Pdpn, Notch1 |
| Bcl3 | 0.0465 | Runx1, Tnfrsf12a |
| Clock | 0.0465 | Serpine1, Bhlhe41 |
| Etv4 | 0.0465 | Mmp3, Plau |

**Supplementary file 1b.**

*(Continued)*

| **Key TF** | **Adjusted *P* value** | **List of overlapped genes** |
| --- | --- | --- |
| Nfkb1 | 1.64E-05 | Cxcl10, Tnfsf13b, Cebpa, Apoe, Cxcl1, Il12a, Myc, Vcam1, Tnf, Sox9, Plin2, Rel, Igf2bp2, Birc3, Hmox1, Nfkbiz, Bcl2 |
| Cebpb | 0.000206 | Bcl2, Myc, Mafb, Cebpa, Btg2, Serpine1, Socs3 |
| Sp1 | 0.000206 | Ces1d, Myc, Mgarp, Serpine1, Sirt1, Cebpd, Egr1, Apoe, Cebpa, Slc2a3, Socs3, Adcyap1r1, Tnf, Ar, Lpl, Bmp4, Igfbp3 |
| Dlx5 | 0.000206 | Wnt5a, Alpl, Myc, Igfbp3 |
| Stat3 | 0.000206 | Mt1, Il12a, Pnp, Socs3, Bcl2, Cebpd, Egr1, Tnf, Myc |
| Rela | 0.000206 | Tnfsf13b, Myc, Vcam1, Igf2bp2, Birc3, Pgf, Sox9, Cxcl10, Apoe, Tnf, Bcl2 |
| Stat1 | 0.000336 | Cxcl9, Egr1, Mt1, Socs3, Cebpd, Cxcl10 |
| Id1 | 0.000336 | Thbs1, Alpl, Rel, Tnf |
| Crebbp | 0.000876 | Socs3, Fosb, Tnf, Hmox1, Bcl2, Mt1 |
| Rel | 0.00115 | Cebpd, Birc3, Il12a, Tnf, Myc |
| Jun | 0.00119 | Plin2, Cebpd, Cxcl1, Serpine1, Hmox1, Aldh1a1, Tnf, Sirt1, Socs3, Plau |
| Hnf1b | 0.00135 | Mafb, Pde4c, Socs3 |
| Hif1a | 0.00324 | Mt1, Cited2, Hmox1, Serpine1 |
| Tfap2a | 0.00381 | Apoe, Cebpa, Meis1 |
| Trp53 | 0.00595 | Btg2, Thbs1, Hmox1, Bcl2, Mt1, Agtr1a, Myc, Serpine1, Egr1, Pappa |
| Ikbkb | 0.00595 | Cxcl9, Cxcl10, Tnf |
| Epas1 | 0.00678 | Serpine1, Cited2, Vcam1 |
| Ppara | 0.00678 | Cebpa, Lpl, Cebpd, Serpine1, Bcl2 |
| Twist1 | 0.00781 | Fgf10, Sox9, Tnf, Mme |
| Smad3 | 0.00809 | Serpine1, Plau, Sirt1, Tnfsf13b, Myc |
| Bcl3 | 0.00899 | Tnf, Cxcl10, Ubc |
| Atf2 | 0.0104 | Sox9, Cebpa, Tnf |
| Fos | 0.0127 | Socs3, Tslp, Mt1, Plau, Egr1 |
| Etv2 | 0.0146 | Sox9, Plau |
| Mtf1 | 0.0146 | Pgf, Mt1 |
| Rbl1 | 0.0146 | Myc, Alpl |
| Ctnnb1 | 0.0167 | Bmp4, Isl1, Myc, Tbx3, Gja1 |
| Elk1 | 0.0181 | Hmox1, Egr1 |
| Fosl1 | 0.0181 | Thbs1, Thbs2 |
| Dnmt3a | 0.0209 | Vtn, Mt1 |
| Foxg1 | 0.0209 | Ebf3, Serpine1 |
| Id3 | 0.0209 | Vcam1, Alpl |
| Tbx1 | 0.0209 | Ripply3, Fgf10 |
| Egr1 | 0.0225 | Tnf, Serpine1, Thbs1, Nr4a1, Il12a |
| Irf1 | 0.0225 | Cxcl10, Il12a, Tnf |
| Foxo3 | 0.0225 | Socs3, Sirt1 |
| Gfi1b | 0.0225 | Meis1, Socs3 |
| Junb | 0.0225 | Plau, Serpine1 |
| Ncoa3 | 0.0225 | Igfbp3, Tbx3 |
| Foxo1 | 0.0242 | Bcl2l11, Egr1, Lpl, Alpl |
| Myc | 0.0242 | Myc, Cebpd, Bcl2, Zfp36 |
| Pparg | 0.0246 | Serpine1, Plin2, Egr1, Hmox1 |
| Nfkb2 | 0.0246 | Myc, Rel |
| Nr4a1 | 0.0246 | Serpine1, Ar |
| Nfe2l2 | 0.0278 | Bcl2, Hmox1, Sult1e1, Mt1 |
| Id2 | 0.0278 | Alpl, Zbtb16 |
| Klf5 | 0.0278 | Egr1, Serpine1 |
| Creb1 | 0.0312 | Bcl2, Nfkbiz, Slc2a3 |
| Runx2 | 0.0336 | Alpl, Thbs1, Hbegf, Sox9 |
| Ets1 | 0.0369 | Egr1, Hmox1, Nr2f1, Bmp4 |
| Ep300 | 0.0382 | Tnfsf13b, Mt1, Hmox1, Socs3 |
| Usf2 | 0.04 | Hmox1, Mt1 |
| Mecp2 | 0.0496 | Slc2a3, Igfbp3 |
| Stat6 | 0.0496 | Tnf, Egr1 |

**Supplementary file 1c.**

| **Gene** | **Secreted** | **GO term related to neuron/glial cell regulation** | **Exclusive expression in activated FAPs** |
| --- | --- | --- | --- |
| **Bdnf** | **O** | **O** | **O** |
| **Apoe** | **O** | **O** | **X** |
| **Ccl2** | **O** | **O** | **X** |
| **Tgfb1** | **O** | **O** | **X** |
| **Tnf** | **O** | **O** | **X** |
| **Vcam1** | **O** | **O** | **X** |
| **Dmp1** | **O** | **X** | **O** |
| **C1qtnf3** | **O** | **X** | **X** |
| **Cxcl1** | **O** | **X** | **X** |
| **Cxcl10** | **O** | **X** | **X** |
| **Il12a** | **O** | **X** | **X** |
| **Il15** | **O** | **X** | **X** |
| **Mmp19** | **O** | **X** | **X** |
| **Mmp3** | **O** | **X** | **X** |
| **Plau** | **O** | **X** | **X** |
| **Plaur** | **O** | **X** | **X** |
| **Serpine1** | **O** | **X** | **X** |
| **Timp1** | **O** | **X** | **X** |
| **Tnfsf13b** | **O** | **X** | **X** |
| **Tslp** | **O** | **X** | **X** |
| **Bcl2** | **X** | **O** | **X** |
| **Egr1** | **X** | **O** | **X** |
| **Egr2** | **X** | **O** | **X** |
| **Hif1a** | **X** | **O** | **X** |
| **Hmox1** | **X** | **O** | **X** |
| **Igf2bp2** | **X** | **O** | **X** |
| **Mt1** | **X** | **O** | **X** |
| **Myc** | **X** | **O** | **X** |
| **Rel** | **X** | **O** | **X** |
| **Sirt1** | **X** | **O** | **X** |
| **Sox9** | **X** | **O** | **X** |
| **Aldh1a1** | **X** | **X** | **X** |
| **Birc3** | **X** | **X** | **X** |
| **Cebpa** | **X** | **X** | **X** |
| **Cebpd** | **X** | **X** | **X** |
| **Itga5** | **X** | **X** | **X** |
| **Nfkbiz** | **X** | **X** | **X** |
| **Pdpn** | **X** | **X** | **X** |
| **Pla2g4a** | **X** | **X** | **X** |
| **Plin2** | **X** | **X** | **X** |
| **Ppp1r13l** | **X** | **X** | **X** |
| **Slc39a14** | **X** | **X** | **X** |
| **Slc8a1** | **X** | **X** | **X** |
| **Socs3** | **X** | **X** | **X** |

**Supplementary file 1d.**

| **No.** | **Primer** | **Usage** | **Sequence (5' to 3')** |
| --- | --- | --- | --- |
| 1 | Prrx1Cre_com_R (P1) | Genotyping PCR | TAG TGA AGT GGA AGT TCC TGG |
| 2 | Prrx1Cre_WT_F (P2) | Genotyping PCR | CAG TTC CTA CCC TGA TTT CC |
| 3 | Prrx1Cre_Tg_F (P3) | Genotyping PCR | GAT CAT AAT CAG CCA TAC CAC |
| 4 | Bdnf_flox_F (P4) | Genotyping PCR | TGT GAT TGT GTT TCT GGT GAC |
| 5 | Bdnf_flox_R (P5) | Genotyping PCR | CGG TTT CTA AGC AAG TGA ACA |
| 6 | Bdnf_recomb_R (P6) | Genotyping PCR | GAA ATT TTC TCC ATC CCT ACT CCG GG |
| 7 | Cre_F | Genotyping PCR | GCA TTA CCG GTC GAT GCA ACG AGT GAT GAG |
| 8 | Cre_R | Genotyping PCR | GAG TGA ACG AAC CTG GTC GAA ATC AGT GCG |
| 9 | Rosa-tdT_WT_F | Genotyping PCR | AAG GGA GCT GCA GTG GAG TA |
| 10 | Rosa-tdT_WT_R | Genotyping PCR | CCG AAA ATC TGT GGG AAG TC |
| 11 | Rosa-tdT_Tg_F | Genotyping PCR | GGC ATT AAA GCA GCG TAT CC |
| 12 | Rosa-tdT_Tg_R | Genotyping PCR | CTG TTC CTG TAC GGC ATG G |
| 13 | Actb_qF | RT-qPCR | CCT CCC TGG AGA AGA GCT ATG |
| 14 | Actb_qR | RT-qPCR | TTA CGG ATG TCA ACG TCA CAC |
| 15 | Ret_qF | RT-qPCR | CCA GGG CTT CCC AAT CAG TT |
| 16 | Ret_qR | RT-qPCR | TTC CAA ACT CGC CTT CTC CC |
| 17 | Gfra1_qF | RT-qPCR | CAC TCC TGG ATT TGC TGA TGT |
| 18 | Gfra1_qR | RT-qPCR | AGT GTG CGG TAC TTG GTG C |
| 19 | Gdnf_qF | RT-qPCR | TCG GCC GAG ACA ATG TAT GA |
| 20 | Gdnf_qR | RT-qPCR | CAA CAT GCC TGG CCT ACT TTG |
| 21 | Bdnf_qF | RT-qPCR | AAG GAC GCG GAC TTG TAC AC |
| 22 | Bdnf_qR | RT-qPCR | CGC TAA TAC TGT CAC ACA CGC |
